# Multiphoton imaging of hippocampal neural circuits: techniques and biological insights into region-, cell-type-, and pathway-specific functions

**DOI:** 10.1117/1.NPh.11.3.033406

**Published:** 2024-03-08

**Authors:** Kotaro Mizuta, Masaaki Sato

**Affiliations:** a RIKEN BDR, Kobe, Japan; b New York University Abu Dhabi, Department of Biology, Abu Dhabi, United Arab Emirates; c Hokkaido University Graduate School of Medicine, Department of Neuropharmacology, Sapporo, Japan

**Keywords:** two-photon microscopy, three-photon microscopy, microendoscopy, calcium imaging, learning and memory, navigation

## Abstract

**Significance:**

The function of the hippocampus in behavior and cognition has long been studied primarily through electrophysiological recordings from freely moving rodents. However, the application of optical recording methods, particularly multiphoton fluorescence microscopy, in the last decade or two has dramatically advanced our understanding of hippocampal function. This article provides a comprehensive overview of techniques and biological findings obtained from multiphoton imaging of hippocampal neural circuits.

**Aim:**

This review aims to summarize and discuss the recent technical advances in multiphoton imaging of hippocampal neural circuits and the accumulated biological knowledge gained through this technology.

**Approach:**

First, we provide a brief overview of various techniques of multiphoton imaging of the hippocampus and discuss its advantages, drawbacks, and associated key innovations and practices. Then, we review a large body of findings obtained through multiphoton imaging by region (CA1 and dentate gyrus), cell type (pyramidal neurons, inhibitory interneurons, and glial cells), and cellular compartment (dendrite and axon).

**Results:**

Multiphoton imaging of the hippocampus is primarily performed under head-fixed conditions and can reveal detailed mechanisms of circuit operation owing to its high spatial resolution and specificity. As the hippocampus lies deep below the cortex, its imaging requires elaborate methods. These include imaging cannula implantation, microendoscopy, and the use of long-wavelength light sources. Although many studies have focused on the dorsal CA1 pyramidal cells, studies of other local and inter-areal circuitry elements have also helped provide a more comprehensive picture of the information processing performed by the hippocampal circuits. Imaging of circuit function in mouse models of Alzheimer’s disease and other brain disorders such as autism spectrum disorder has also contributed greatly to our understanding of their pathophysiology.

**Conclusions:**

Multiphoton imaging has revealed much regarding region-, cell-type-, and pathway-specific mechanisms in hippocampal function and dysfunction in health and disease. Future technological advances will allow further illustration of the operating principle of the hippocampal circuits via the large-scale, high-resolution, multimodal, and minimally invasive imaging.

## 1 Introduction

The hippocampus plays a pivotal role in memory and navigation. Historically, this concept was established through two streams of research. First, a prominent patient, H.M., who underwent bilateral resection of the medial temporal lobe including the hippocampus to treat intractable epilepsy in the 1950s, failed to form postoperative learning and lost memory of events in the years prior to surgery; however, his childhood memories remained normal.^1^ Second, the discovery of place cell activity in the rodent hippocampus has revealed that hippocampal cells are activated when the animal is in a particular place.^2^ Although these two lines of study have been conducted as relatively independent research areas for many years, efforts to reconcile them have been vigorously pursued in recent years.^3^^,^^4^

The recording of hippocampal place cells is typically conducted electrophysiologically. In recent years, however, researchers have also started to incorporate optical recordings through imaging techniques. Two-photon excitation fluorescence microscopy using near-infrared ultrashort pulsed laser has large depth penetration into highly light-scattering biological tissues and was introduced into neuroscience in the 1990s.^5^ Since then, it has become a powerful tool for high resolution imaging of neural circuit structure and function in the living brain. Two-photon imaging paved the way for many new insights into the neural circuits in brain regions that can be easily observed below the surface of the brain such as the cerebral cortex. However, optical observation of the pyramidal cell layer of the dorsal CA1, the neuronal layer closest to the brain surface in the mouse hippocampus, requires more sophisticated techniques. This is because CA1 pyramidal neurons are located more than 1 mm deep from the brain surface in mature mice, beneath the depths of the cerebral cortex, the corpus callosum, and the stratum oriens of the hippocampus.

In this review, we first briefly outline the three major techniques for multiphoton imaging of the hippocampus: (1) implantation of imaging cannulae, (2) microendoscopy, and (3) use of long-wavelength light sources. We then discuss its advantages and disadvantages compared to other recording methods, as well as its key innovations. The experimental and analytical practices of this technique will also be explained.

## 2 Hippocampal Imaging Techniques

### 2.1 Imaging Cannula Implantation

**Fig. 1:**
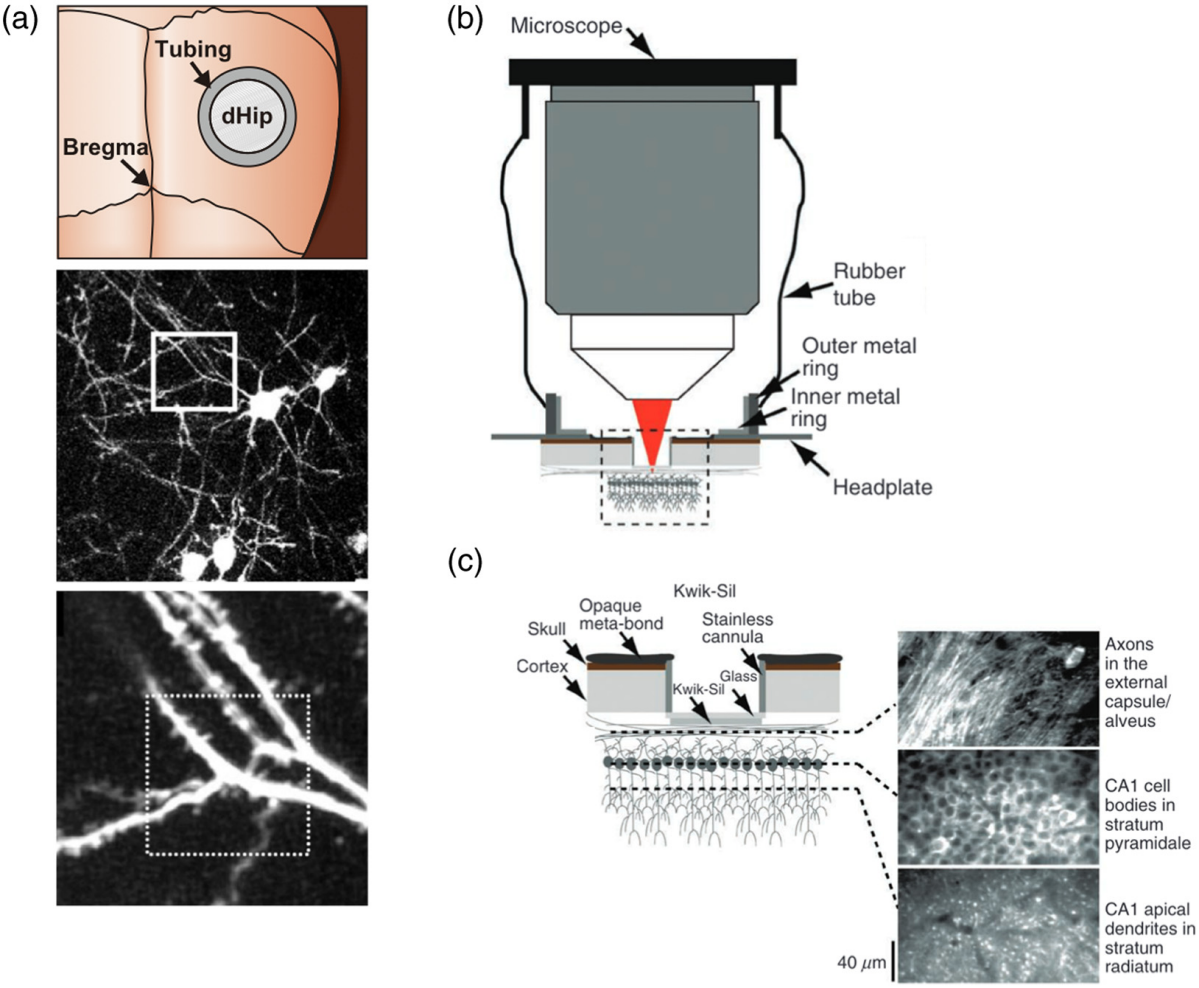
Imaging hippocampal neurons through an implanted cannula. (a) Implantation of an acrylic imaging cannula onto the dorsal CA1 hippocampus (dHip) (top), an image of GFP-labeled CA1 pyramidal neurons (middle), and an enlarged view of the enclosed dendrites (bottom). Adapted from Mizrahi et al.^6^ Copyright 2004 Society for Neuroscience. (b) A cross-section view of two-photon imaging of CA1 neurons through an imaging cannula. The rubber tube is used to prevent stray light from a virtual reality screen. (c) A close-up view of the area enclosed with dashed line in panel (b) and images of the CA1 area acquired at different focal depths. Panels (b) and (c) are adapted with permission from Dombeck et al.^7^

The technique of aspiration and removal of portions of brain tissue has long been used in brain physiology experiments. Employing this method, Mizrahi et al. performed the first-ever two-photon imaging of hippocampal CA1 pyramidal cell spines in living mice through implantation of a ring-shaped window after suction removal of the cerebral cortex overlying the dorsal CA1 [Fig. 1(a)].^6^ Dombeck et al. used a similar technique to perform calcium imaging of the CA1 neurons in mice navigating in a virtual reality (VR) environment and revealed that hippocampal place cell activity could be imaged under head fixation [Figs. 1(b) and 1(c), see also Fig. 3(a)].^7^ Since then, this technique has been adopted by many laboratories. This method is relatively invasive but has the advantage of providing excellent optical conditions for observation. Therefore, it has been also used for imaging calcium responses in fine cellular structures such as dendrites and axons (see Secs. 3.3 and 3.4 on dendrite and axon imaging, respectively).^8^^,^^9^ Moreover, the integration of transparent graphene microelectrodes with imaging cannula allows simultaneous long-term electrical recording with two-photon calcium imaging from the same CA1 neuronal populations.^10^

### 2.2 Microendoscopy

**Fig. 2:**
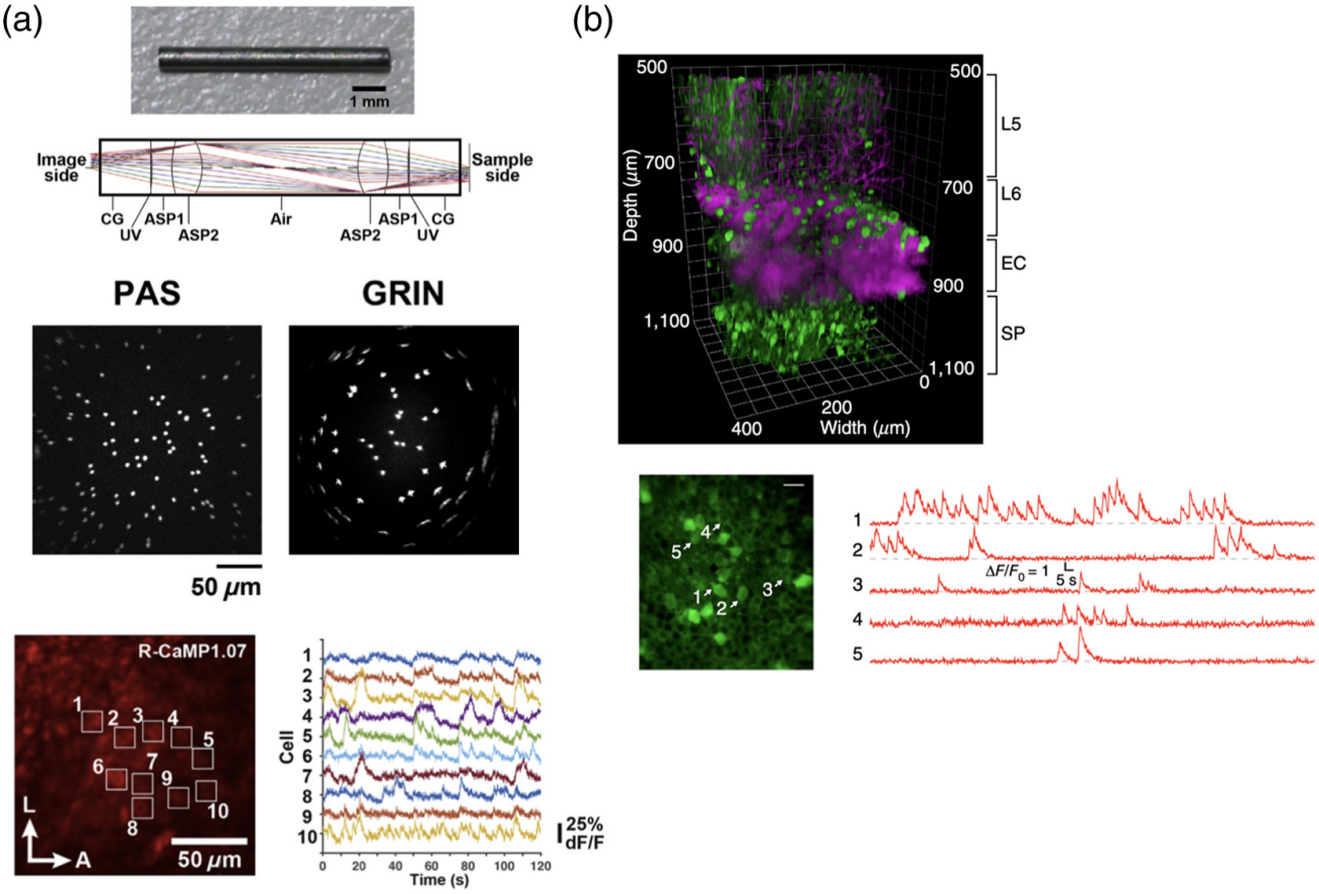
Microendoscopy and the use of long-wavelength light source for hippocampal imaging. (a) Design of the aspherical lens-based microlens probe (top). Images of fluorescent microspheres obtained using the aspherical lens-based microlens probe (PAS, middle left) and GRIN lens (GRIN, middle right). Two-photon imaging of R-CaMP 1.07-labeled CA1 pyramidal neurons through the aspherical microlens probe (bottom left) and time-varying R-CaMP fluorescence signals of 10 selected neurons (bottom right). Adapted from Sato et al.^15^ (b) Three-dimensional view of the intact cortex (L5 and L6) and dorsal CA1 hippocampus (SP) reconstructed from three-photon imaging of GCaMP6s-labeled CA1 pyramidal neurons (top). Three-photon imaging of GCaMP6s-labeled CA1 pyramidal neurons (bottom left) and time-varying GCaMP6s fluorescence signals of six selected neurons (bottom right). Adapted with permission from Ouzounov et al.^19^

Deep-brain imaging is also made possible by embedding micro-optical components that relay deep images to surfaces that can be reached by microscope lenses. Typical examples include gradient refractive index (GRIN) lenses with a radial refractive index gradient and diameters of approximately 0.5 to 1.5 mm. By implanting these elongated lens probes into the brain, deep-brain regions can be endoscopically imaged when combined with one-photon or two-photon fluorescence microscopy.^11^^,^^12^ Combined with miniaturized head-mounted one-photon microscopes, imaging of deep-brain activity under freely moving conditions is also being actively conducted.^13^ Unlike the other two approaches for deep brain imaging (Secs. 2.1 and 2.3), microendoscopy has the advantage of imaging cells at depths beyond the working distance of the microscope objective (typically up to 3 to 4 mm). One drawback, however, is that once the lens probe is implanted in the brain, only cells in the probe tip vicinity can be observed in standard microendoscopic imaging. To overcome this limitation, a recent study reported a two-photon microendoscope that can rapidly change the focal plane by combining an electrically tunable lens (ETL) with a GRIN lens.^14^ Another technical issue is that the GRIN lens has strong inherent monochromatic and chromatic aberrations, posing potential problems such as narrowing of the field of view (FOV), image quality degradation, and experimental flexibility limitation. To address this issue, non-GRIN-lens microendoscopic probes made with aspherical lenses have been shown to effectively eliminate these aberrations [Fig. 2(a)].^15^

### 2.3 Long-Wavelength Light Source

The longer the wavelength of the excitation light of a fluorescent substance, the less likely it is to be scattered, and the deeper the light penetrates into tissues. Taking advantage of this fact, significant progress has been made in the development of techniques for imaging hippocampal neurons from the surface of the brain without removing the cerebral cortex. Kawakami et al.^17^ used a 1030-nm laser to image hippocampal CA1 pyramidal cells labeled with yellow fluorescent protein (YFP) from the brain surface of a young 4-week-old mouse. Kondo et al.^18^ showed that calcium imaging of hippocampal CA1 pyramidal cells from 4-week-old mice labeled with the red fluorescent calcium sensor protein jRGECO1a can be conducted from the brain surface via two-photon excitation using a 1100-nm narrow laser beam. These two studies utilized two-photon imaging, but recent studies have also used three-photon imaging. Three-photon imaging significantly increases both the signal-to-background ratio and imaging depth for high-resolution fluorescence imaging. These traits make it promising as a minimally invasive imaging method for hippocampal imaging. For example, Horton et al.^16^ imaged hippocampal CA1 pyramidal cells labeled with red fluorescent protein (RFP) from the brain surface of 6-week-old mice through three-photon excitation with a 1700-nm laser. In a separate study, Ouzounov et al.^19^ succeeded in calcium imaging of hippocampal CA1 pyramidal cells from 11- to 16-week-old mature mice labeled with GCaMP6s by three-photon imaging from the brain surface [Fig. 2(b)]. These studies highlight the more advanced capabilities of three-photon imaging. However, it is currently not as widely used as two-photon imaging, likely due to the high cost and low availability of suitable light sources Recently, commercial suppliers have started producing lasers and microscopes that are compatible with three-photon imaging. This can potentially make this particular imaging technique more popular in the future.

### 2.4 Advantages and Drawbacks of Multiphoton Calcium Imaging

Before optical monitoring of neuronal activity became possible, recordings of hippocampal neural activity in living animals were performed primarily using electrophysiological techniques.^20^ Advantages of multiphoton calcium imaging over conventional electrophysiology include: the ability to see cells and cellular structures being recorded on a high spatial resolution image; the ability to record almost comprehensively from hundreds to thousands of cells in the FOV, including those with low activity or in close proximity to each other; the ability to record specifically from genetically defined cell types, including non-spiking cell types such as glial cells; the ability to record specifically from fine subcellular compartments, such as axons and dendrites, and the ability to monitor not only neuronal activity but also the dynamics of specific neurotransmitters by using fluorescent biosensor molecules.^21^ In Section 3 below, we summarize some biological findings obtained by taking advantage of these aspects of multiphoton imaging.

On the other hand, multiphoton calcium imaging has the drawback of low temporal resolution. In general, calcium imaging indirectly observes neuronal activity by monitoring the fluorescence changes of calcium indicator molecules associated with the relatively slow increase in intracellular calcium concentration following electrical activity. In addition, because standard multiphoton microscopy typically acquires images by scanning the focal point where multiphoton excitation occurs on a pixel-by-pixel basis, its imaging speed is at most tens of images per second. In the hippocampus, characteristically fast electrical activity such as sharp-wave ripples (SWR), replay, and oscillatory population activities of various frequencies are known to occur.^22^^,^^23^ However, it is technically challenging to precisely record these phenomena with calcium imaging for the above reasons. Membrane voltage imaging using genetically-encoded voltage indicators may be able to record these fast dynamics.^21^^,^^24^ These indicators can provide optical traces of membrane potential dynamics with submillisecond spike timing accuracy. Although currently limited to recording a small number of neurons,^24^^–^^27^ more responsive, more photostable, and multiphoton excitable voltage sensors^28^^–^^31^ in combination with a microscope with fast imaging speed^32^^–^^34^ should enable long-duration optical recording of electrical activity in deep and large neuronal populations with high spatial resolution.

### 2.5 Key Innovations and Experimental and Analytical Practice in Multiphoton Hippocampal Imaging

**Fig. 3:**
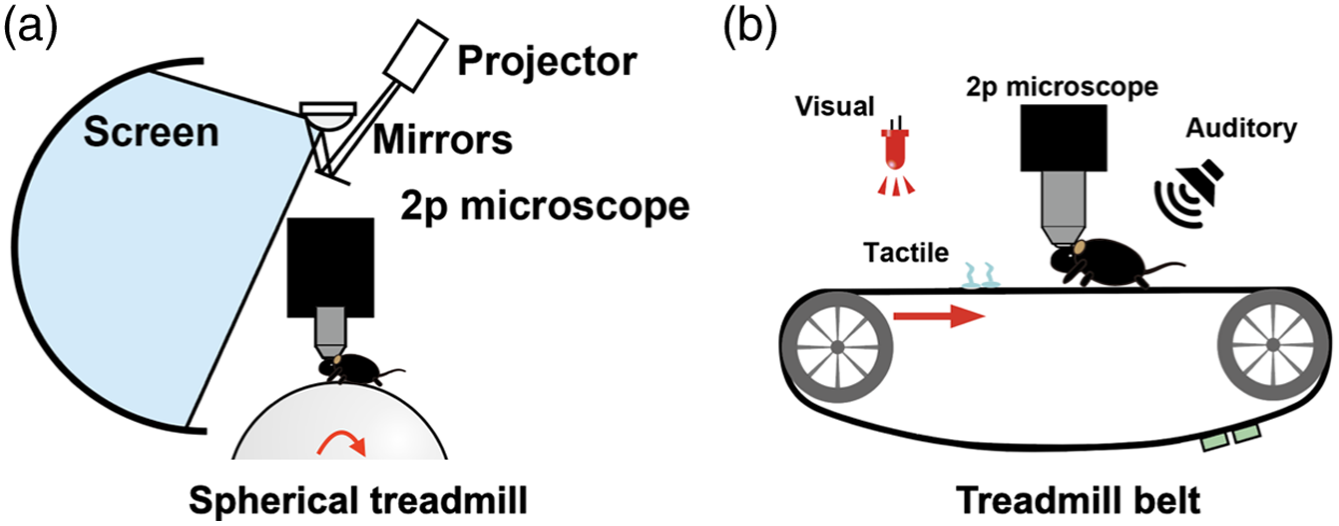
Behavior apparatus used to image hippocampal place cell activity. (a) A toroidal screen and spherical treadmill-based VR system. (b) A multimodal treadmill belt system.

Multiphoton imaging of hippocampal place cell activity has been made possible by a combination of innovative techniques. In addition to the techniques for imaging hippocampal cells described above, multiphoton imaging of place cell activity requires animals to perform navigational behaviors with their heads fixed under a microscope. For this purpose, head-fixed multiphoton imaging typically uses a VR environment in which a landscape projected on an immersive screen moves in tandem with the animal’s movement^7^ [Fig. 3(a)], or a multimodal belt treadmill in which multiple types of sensory stimuli are delivered in a location-specific manner^8^ [Fig. 3(b)].

Innovative optical technologies that were proven to work in other brain regions have also been applied to multiphoton imaging of hippocampal functional circuits. As shown below, prominent examples include simultaneous two-photon single cell optogenetics and calcium imaging to study place field formation mechanisms,^35^ multiplane imaging with an ETL for simultaneous imaging of pyramidal cell dendrites and cell bodies,^9^ two-photon super-resolution microscopy for measuring turnover of hippocampal dendritic spines,^36^ and random-access microscopy for fast three-dimensional (3D) imaging of large-scale interneuron dynamics.^37^

To date, most advanced imaging techniques have often been developed using the cerebral cortex as a model system. The hippocampus is located deep beneath the cortex, and two-photon imaging of this area typically requires the use of a cannula embedded in the cortex to obtain hippocampal fluorescent signals. This makes hippocampal imaging optically disadvantageous compared to cortical imaging. Because of this, some innovative multiphoton microscopy techniques have not yet been applied to the imaging of functional circuits in the hippocampus. For example, the CA1 area is not anatomically homogeneous, and is known to have different features along the distal-proximal and nasal-temporal axes.^38^ Therefore, comprehensive mapping of large areas of CA1 and other subfields with wide-field multiphoton microscopy^39^^–^^41^ might reveal previously unknown large-scale functional organization. Since implanting a larger cannula for wide-field observation could be more invasive, it may be necessary to image the hippocampus through the intact cortex using three-photon imaging.^42^

There are several key points to obtain high quality data in multiphoton imaging of the hippocampus. First, a successful cannula implantation is essential (for step-by-step protocol, see Ulivi et al.^43^). Implanting a cannula requires removing a part of the cerebral cortex, making the surgery invasive. In our experience, animals need to recover for at least 3 to 4 weeks after surgery for stable chronic recording. When imaging the dorsal CA1 field in mice, the pyramidal cell layer is approximately 150  μm below the surface of the hippocampus. This makes it less difficult to observe their cell bodies with a standard two-photon microscope. Although we prefer to use transgenic mice because numerous pyramidal cells within an FOV are uniformly labeled with genetically encoded calcium indicators,^44^^–^^47^ many studies also use labeling with adeno-associated viral vectors. When observing well-isolated fine subcellular structures, such as individual dendrites, sparse cell labeling obtained by co-infection of driver- and reporter-expressing viral vectors may be necessary.^9^^,^^48^

Analysis pipelines for calcium imaging data generally consists of motion correction, extraction of neurons’ locations and their time-varying fluorescence traces (source extraction), spike inference from the fluorescence traces, and registration across multiple sessions if animals are imaged repeatedly (for details, see Pnevmatikakis^49^). Imaging of hippocampal place cells involves locomotion of the animal. Motion artifacts can be corrected offline or online to some degree,^50^^–^^54^ but it is important to make an effort to experimentally minimize brain motion.^7^^,^^51^ There are several algorithms for source extraction and spike inference.^49^ Currently, non-negative matrix factorization-based methods are commonly used.^55^^–^^58^ The pyramidal cell layer of the hippocampus is densely populated with neurons, and these methods can effectively separate spatially overlapping cell activities. When the technology that records cells becomes more advanced and eventually increases the number of cells that can be recorded at a time, more efficient image analysis methods are required.^59^^,^^60^

## 3 Biological Insights Obtained From Multiphoton Imaging of Hippocampal Circuits

In this section, the findings on the operating principles of hippocampal neural circuits obtained from multiphoton imaging by region, cell type, and cell compartment are reviewed (Table 1).

**Table 1:** Selected studies of multiphoton imaging of hippocampal neural circuits.

Region	Cell type	Compartment	Fluorescent reporters	Genetic and viral labeling	Disease model	Behavioral task	Setup and conditions	References
CA1	PC	Soma	GCaMP3	AAV(Syn::GCaMP3)		RL	VR	Dombeck et al.^7^
CA1	PC	Soma	GCaMP6f	AAV(CaMKII::GCaMP6f)		RL	BT	Danielson et al.^61^
CA1	PC	Soma	GCaMP3	Tg (Thy1::GCaMP3)		RL	VR	Gauthier and Tank^62^
CA1	PC	Soma	G-CaMP7	Tg(Thy1::G-CaMP7)	ASD (Shank2 KO)	RL	VR	Sato et al.^45^
CA1	PC	Soma	G-CaMP7	Tg(Thy1::G-CaMP7)		RL	VR	Mizuta et al.^46^
CA1	PC	Soma	GCaMP6f	Tg(Thy1::GCaMP6f)		Navigational DM	VR	Nieh et al.^63^
CA1	PC	Soma	Oregon Green BAPTA-1 AM	N/A		TC	HF	Modi et al.^64^
CA1	PC	Soma	TurboFP635 and d2Venus	AAV(E-SARE::d2Venus-cHS4-ePGK::TurboFP635)		Novel context exposure	AN	Attardo et al.^65^
CA1	PC	Soma	GCaMP6f	Tg(CaMKII::Cre) and AAV(Syn::FLEX-GCaMP6f)		TC	HF	Ahmed et al.^66^
CA1	PC and GAD+ IN	Soma	GCaMP6f and tdTomado (GABAergic neurons)	AAV(Syn::GCaMP6f) and Tg(GAD2::Cre, ROSA-CAG::LCL-tdTomato)		Olfactory DNMS	HF	Taxidis et al.^67^
CA1	PC	Soma	GCaMP6f and jGCaMP8m	AAV(Syn::GCaMP6f, Syn::jGCaMP8m)		Olfactory TC	HF, CT	Biane et al.^68^
CA1	PC and GAD+ IN	Soma	GCaMP6f and tdTomado (GABAergic neurons)	Tg(GAD1::Cre) and AAV(Syn::GCaMP6s, CAG::FLEX-tdTomato)		N/A	HF	Dard et al.^69^
CA1	PC	Soma	GCaMP6s	Tg(Emx1::Cre, ROSA-CAG::LCL-GCaMP6s)		N/A	AN	Graf et al.^70^
CA1	PC	Soma	GCaMP6f	AAV(Syn::GCaMP6f)		Olfactory-guided navigation	VR	Radvansky and Dombeck^71^
CA1	PC	Soma	GCaMP6f	AAV(Syn::GCaMP6f)		RL	VR	Radvansky et al.^72^
CA1	PC	Soma	GCaMP6f	Tg(Thy1::GCaMP6f)		Olfactory-guided navigation	BT	Zemla et al.^73^
CA1	PC, PV+ IN, and SOM+ IN	Soma	GCaMP6f(SOM+ IN, PV+ IN) and jRGECO1a (all neurons)	Tg(SOM::Cre, PV::Cre) and AAV(Syn::FLEX-GCaMP6f)		RL	VR	Arriaga and Han^74^
CA1	PV+ IN and SOM+ IN	Soma	GCaMP6f	Tg(SOM::Cre, PV::Cre) and AAV(Syn::FLEX-GCaMP6f)		RL	VR	Arriaga and Han^75^
CA1	VIP+ IN	Soma	GCaMP6f, ArchT-tdtomato, ChR2-tdTomato or GFP (VIP+ IN), and GCaMP6f (all neurons)	Tg (VIP-Cre), AAV(EF1α::(GCaMP6f)Cre, Syn::(ArchT-tdTomato)Cre, Syn::(ChR2-GFP)Cre, Syn::(GFP)Cre, Syn::(tdTomato)Cre, CaMKII::GCaMP6f, EF1α::(bReaChES-tdTomato)Cre, and EF1α::((ArchT-tdTomato)Cre)		RL	BT	Turi et al.^76^
CA1	VIP+ IN	Soma	GCaMP6f	Tg(VIP::Cre, ROSA-CAG::LCL-tdTomato) and AAV(Syn::FLEX-GCaMP6f)		Running	CT	Luo et al.^77^
CA1	PV+ IN	Soma	GCaMP6f	Tg (PV-Cre) and AAV(CAG::FLEX-GCaMP6f)		Water licking	HF	Judák et al.^78^
CA1	VIP+ IN and VGAT+ IN	Soma	GCaMP6f	Tg(VGAT::Cre, VIP::Cre) and AAV(Syn::FLEX-GCaMP6f, Syn::((GCaMP6f)Cre)		RL	BT	Geiller et al.^37^
CA1	PC and GAD+ IN	Soma	GCaMP5G and GCaMP6m	Tg(GAD67::Cre) and AAV(Syn::GCaMP5G, Syn::GCaMP6m, Syn::FLEX-GCaMP5G)	Epilepsy (pilocarpine)	Running	BT	Muldoon et al.^79^
CA1	AC	Soma	GCaMP6s	Tg(GFAP::CreERT2, ROSA-CAG::LCL-GCaMP6s)		Elevated plus maze	VR	Cho et al.^80^
CA1	AC and PC	Soma	GCaMP6f (AC) and jRCaMP1a(PC)	AAV(gfaABC1D::cyto-GCaMP6f, CaMKII::jRCaMP1a)		RL	VR	Curreli et al.^81^
CA1	AC, PV+ IN, and SOM+ IN	Soma	cytoGCaMP6f(AC) and tdTomato (SOM+ IN and PV+ IN)	Tg(PV::Cre, SST::Cre, ROSA-CAG::LCL-tdTomato) and AAV(gfaABC1D::cyto-GCaMP6f)		RL	VR	Doron et al.^82^
CA1	PC	Soma	GCaMP5G	AAV(Syn::GCaMP5G)		Running	BT	Villette et al.^83^
CA1	PC	Soma	GCaMP5G, GCaMP6m, and GCaMP6f	AAV(Syn::GCaMP5G, Syn::GCaMP6m, Syn::GCaMP6f)		RL	BT	Malvache et al.^84^
CA1	PC	Soma	Fluo-8 AM	N/A	AD (APP/PS1)	N/A	AN	Busche et al.^85^
CA1	SOM+ IN	Soma, dendrite, and axon	GCaMP6m (PC) and GFP (SOM+ IN)	Tg(Gad1::GFP) and AAV(Syn::GCaMP6m)	AD (APP/PS1)	Air puff stimulation	Spherical treadmill	Schmid et al.^86^
CA1	PC	Soma	jRGECO1a and GFP	Tg(c-fos::GFP) and AAV(CaMKII::Cre, Syn::FLEX-jRGECO1a)	AD (APP/PS1)	Novel context exposure	BT	Poll et al.^87^
CA1	PC	Soma	G-CaMP7	Tg(Thy1::G-CaMP7)	AD (APPNL-G-F)	RL	VR	Takamura et al.^47^
CA1	PC	Soma	GCaMP6f	AAV(Syn::GCaMP6f)	SZ (Df(16)A^+/−^)	RL	BT	Zaremba et al.^88^
CA1	PC	Soma	GCaMP3 and C1V1	Tg(Thy1::GCaMP3) and AAV(CaMK2::C1V1(E122T/E162T)-EYFP)		RL	VR	Rickgauer et al.^35^
CA1	PC	Soma	GCaMP6f, mCherry, and C1V1	AAV(Syn::GCaMP6f, CaMKII::C1V1(E162T)-mCherry)		RL	VR	Robinson et al.^89^
CA1	PC	Dendrite	GFP	Tg (Thy1::GFP (O line))		N/A	AN	Mizrahi et al.^6^
CA1	PC	Dendrite	GFP and YFP	Tg (Thy1::GFP (M line), Thy1::YFP (H line))		N/A	AN	Pffeifer et al.^36^
CA1	PC	Dendrite	YFP	Tg (Thy1::YFP (H line))		N/A	AN	Gu et al.^90^
CA1	PC	Dendrite	GCaMP6f	AAV(CaMKII–Cre, Syn::FLEX–GCaMP6f)		RL	VR	Sheffield and Dombeck^9^
CA1	PC, SOM+ IN, and PV+ IN	Soma, dendrite, and axon	GCaMP6f	Tg (PV::Cre, SOM::Cre) and AAV(CaMKII::Cre, Syn::FLEX-GCaMP6f, Syn::FLEX-GCaMP6s)		RL	VR	Sheffield et al.^91^
CA1	PC	Dendrite	iGluSnFR and jRGECO1a	AAV(CaMKII::Cre, Syn::FLEX-SF-iGluSnFR-A184S, Syn::FLEX-NES-jRGECO1a)		RL	VR	Adoff et al.^48^
CA1	PC	Dendrite	GCaMP6f	AAV(Syn::GCaMP6f)Cre and IUEP(CAG::Cre)		Random foraging	BT	Rolotti et al.^92^
CA1	MS axon	Axon	GCaMP5s (MS GAD+ neurons) and tdTomato (CA1 GAD+ IN, PV+ IN, SOM+ IN)	Tg (Gad65::Cre, PV::Cre, SOM::Cre, ROSA-CAG::LCL-tdTomato) and AAV(Syn:GCaMP5)		Aversive learning	BT	Kaifosh et al.^8^
CA1	PV+ IN	Axon	GCaMP5 and tdTomato (PV+ IN)	Tg(PV::Cre, ROSA-CAG::LCL-tdTomato) and AAV(Syn::(GCaMP5)cre)		Running	BT	Lee et al.^93^
CA1	MRN axon	Axon	GCaMP6f	Tg (Sert::Cre) and AAV(CAG::FLEX-GCaMP6f)		RL	VR	Luchetti et al.^94^
CA1	PC and LC axon	Soma and axon	GCaMP6f(PC) and GCaMP6s(LC)	Tg (TH::Cre, VIP::Cre), AAV(EF1a::(bReaChes-tdTomato)Cre, EF1a::(GCaMP6s)Cre, Syn::GCaMP6f, and CaMKII::GCaMP6f)		RL	BT	Kaufman et al.^95^
CA1/CA3/DG	PC, GC, and MEC axon	Soma and axon	GCaMP6s	AAV(Syn::GCaMP6s)		RL	VR	Cholvin et al.^96^
CA1	CA3 axon	Axon	GCaMP6f	Tg(Grik4::Cre) and AAV(Syn::FLEX-GCaMP6f)		RL	BT	Terada et al.^97^
DG	GC	Soma	R-CaMP1.07 and GCaMP6s	Tg (Rbp4::Cre) and AAV(EF1a::DIO-R-CaMP1.07, CAG::FLEX-GCaMP6s)		Running	CT	Pilz et al.^98^
DG	GC	Soma	GCaMP6f (all GC) and tdTomato (abGC)	Tg (Nestin::CreERT2, ROSA-CAG::LCL-tdTomato) and AAV(Syn::GCaMP6f)		RL	BT	Danielson et al.^99^
DG	GC	Soma	GCaMP6f (all GC) and tdTomato (abGC)	Tg (Nestin::CreERT2, ROSA-CAG::LCL-tdTomato) and AAV(Syn::GCaMP6f)	Epilepsy (kainic acid)	Running	BT	Sparks et al.^100^
DG	Mossy cell	Soma	GCaMP6f	AAV(CAG::Cre, Syn::FLEX-GCaMP6f)		RL	BT	Danielson et al.^101^
DG/CA3/CA1	GC, PC, and PV+ IN	Soma	GCaMP6f and tdTomato (PV+ IN)	Tg (PV::Cre, ROSA-CAG::LCL-tdTomato) and AAV(Syn::GCaMP6f, CAG::GFP)		RL	VR	Hainmueller and Bartos^102^
DG	GC	Soma	GCaMP6s	AAV(Syn::GCaMP6s)		RL	VR	Cholvin and Bartos^103^
DG	GC and MEC axon	Soma and axon	GCaMP6s (GC) and jRGECO1a (MEC axon)	Tg (Thy1::GCaMP6s) and AAV(CaMKIIa::NES-jRGECO1a)		Running	BT	Pofahl et al.^104^
DG	GC	Soma	GCaMP6s	AAV(Syn::GCaMP6s)		Spatial cue task	BT	Tuncdemir et al.^105^
DG	NSC	Soma	tdTomato	Tg (Ascl1::CreERT2, ROSA-CAG::LCL-tdTomato)		N/A	AN	Pilz et al.^106^
DG	NSC	Soma	tdTomato	Tg (Gli1::CreERT2, Ascl1::CreERT2 and ROSA-CAG::LCL-tdTomato)		N/A	AN	Bottes et al.^107^
DG	GC and AC	Soma	jGCaMP7s (GC) and RFP (AC)	Tg (GFAP::RFP) and AAV(Syn::jGCaMP7s)		N/A	AN	Schneider et al.^108^

Abbreviations: AAV, adeno-associated virus; abGC, adult-born GC; AC, astrocytes; AD, Alzheimer’s disease; AN, anesthesia; ASD, autism spectrum disorder; BT, belt treadmill; CT, cylindrical treadmill; DM, decision making; DNMS, delayed non-matching to sample; GAD+, glutamic acid decarboxylase-positive; GC, granule cells; HF, head fixation without treadmill; IN, interneurons; IUEP, *in utero* electroporation; KO, knockout; LC, locus coeruleus; MEC, medial entorhinal cortex; MRN, median raphe nucleus; MS, medial septum; NSC, neural stem cells; PC, pyramidal cells; PV+, parvalbumin-positive; RL, reward learning; SOM+, somatostatin-positive; SZ, schizophrenia; TC, trace conditioning; Tg, transgenic mice; VGAT+, vesicular GABA transporter-positive; VIP+, vasoactive intestinal polypeptide-positive; and VR, virtual reality.

### 3.1 CA1

#### 3.1.1 Pyramidal neurons

**Fig. 4:**
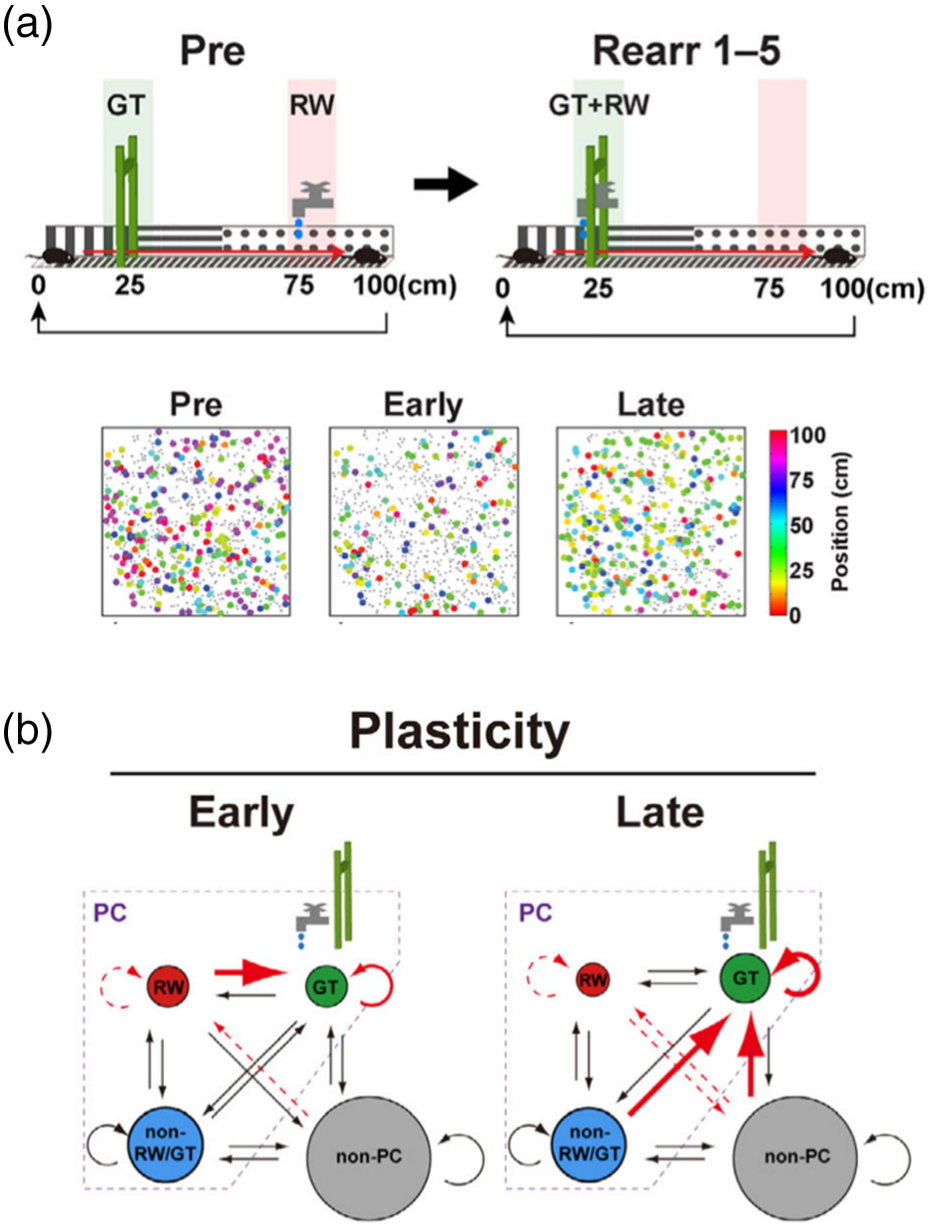
Plasticity mechanism for the CA1 cognitive maps. (a) CA1 map reorganization induced by reward relocation. Mice were first trained on the standard linear track (Pre). Once training was completed, mice were retrained in a new arrangement in which the location of reward delivery was shifted to match the location of the landmark (Rearr 1–5) (top). Examples of place cell maps imaged in the same animal in the preceding control (immediately before the reward relocation), early (first retraining session), and late (fifth retraining session) phases of the reward-rearrangement task (bottom). (b) A model for CA1 map plasticity. Immediately after reward relocation, the fraction of place cells decreases due to decreased reward cell formation and stabilization, and a subpopulation of reward cells moves their fields to the new reward location (early). After a few sessions, the stability and formation of reward+landmark cells increase, as do recruitment from the outside place cells to reward+landmark cells (late). Adapted from Mizuta et al.^46^

After the first imaging of the CA1 place cell activity,^7^ calcium imaging of CA1 pyramidal neurons during behavior has been widely reported. Imaging demonstrated that pyramidal neurons in superficial sublayer form more stable place maps, whereas those in deep sublayer are preferentially modulated during goal-oriented learning.^61^ Imaging of the CA1 neuron activity during reward location alternation task in VR identified a subset of pyramidal cells that are involved in reward coding.^62^ Hippocampal place maps display a phenomenon called over-representation, in which place cells accumulate at locations with behaviorally relevant features such as reward and landmarks. Calcium imaging of CA1 pyramidal neurons in mice navigating a virtual linear track with reward and a landmark placed at distinct locations demonstrated that over-representation of these locations occurs with different time courses and molecular mechanisms involving the synaptic scaffold protein Shank2 and primarily owing to selective stabilization and accumulation of relevant place cells.^45^ Additionally, when mice were retrained under a new condition in which the reward was shifted to match the landmark location [Fig. 4(a)], a subset of place cells that were active at the initial reward site shifted their place fields to the new reward site, along with delayed recruitment of nearby place cells [Fig. 4(b)].^46^ Collectively, these findings reveal a basic comprehensive picture of cellular dynamics associated with hippocampal cognitive map formation and plasticity.

Recently, an olfactory VR system, using rapid flow controllers and an online algorithm to produce precise odorant distributions, demonstrated that odor-guided virtual navigation behavior engages CA1 place cells similar to those found in visual virtual environments.^71^ They further revealed that mice navigate the same multisensory environment using either vision, odor, or both, and hippocampal spatial mapping relies on the modality used for navigation.^72^ An odor-cued navigation task also exhibited that mice develop different neuronal spatial representations depending on task rules.^73^ Thus, multisensory VR environments and navigation tasks that depend on non-visual modalities widen a potential for investigating mechanisms underlying hippocampal sensory-spatial and memory processing.

While hippocampal neurons encode physical variables such as space, they can also encode more abstract cognitive variables such as learned knowledge. CA1 neurons jointly encode accumulated evidence with spatial position as demonstrated by two-photon calcium imaging of CA1 pyramidal neurons in mice performing a decision-making task in a virtual T-maze.^63^ This suggests that the hippocampus creates task-specific low-dimensional neural manifold that contain a geometric representation of learned knowledge.

Donald O. Hebb, a Canadian psychologist, postulated the concept of “cell assembly” as a group of neurons that are repeatedly coactivated and support various cognitive processes, including memory. Hippocampal neuronal populations are known to show structured activity, such as sequential activity and synchronous activity, even under spatial cue-deprived environments, and two-photon imaging of these preconfigured neuronal ensemble activity has provided important insights into their organization. Villette et al.^83^ imaged the activity of CA1 pyramidal cell populations while mice ran on a treadmill without external cues and rewards. They found that the dynamics of a hippocampal population code spontaneously displays recurring sequences in the presence of self-motion cues, suggesting that such a functional structure may be an internal cognitive template that shapes behavior. In their follow-up study, Malvache et al.^84^ conducted simultaneous two-photon calcium imaging with a contralateral local field potential (LFP) recording to investigate patterns of synchronous activation of the CA1 ensembles in mice alternating running and rest on a treadmill. They observed that anatomically intermingled and functionally orthogonal assemblies undergo recurrent reactivation. Additionally, these assemblies represented discrete temporal segments of neuronal sequences observed during runs, and multiple assemblies could be combined into longer chains of replay.

In recent years, some studies have combined optogenetics with multiphoton imaging. However, they are typically manipulations that stimulate or inhibit brain regions outside the FOV^94^^,^^95^ or full field stimulation or inhibition of a particular neuronal subtype within the FOV.^76^ Thus far, limited studies have conducted imaging while optogenetically manipulating neuronal activity at the single cell level within the FOV. Rickgauer et al.^35^ conducted such an experiment first. Technically, they performed dual-wavelength two-photon excitation to observe the activity of GCaMP3-labeled CA1 pyramidal neuron populations at 920 nm and photostimulate neurons expressing a red-shifted optogenetic actuator C1V1(E122T/E162T) in the same FOV at 1064 nm in mice exploring a virtual environment. They manipulated task-modulated activity of the CA1 place cells by artificially biasing natural place-field activity and revealed subthreshold dynamics and local interactions between multiple neurons underlying place field formation using this system. Recently, light modulator-mediated two-photon holographic optogenetics that allows simultaneous activation of several place cells has also been developed.^89^ Targeted stimulation of specific place cell ensembles biased the spatial behavior of mice in a virtual environment, thereby providing a direct causal evidence linking place cell activity and spatial navigation.

Finally, two-photon calcium imaging of CA1 pyramidal neurons during non-spatial tasks^64^^–^^68^ and that of developing CA1 hippocampus in living mice^69^^,^^70^ have provided valuable knowledge into their implication in learning and memory, as well as in postnatal development of functional hippocampal circuits.

#### 3.1.2 Inhibitory interneurons

In the hippocampus, network excitation computation and plasticity are controlled through inhibition. ETL-mediated multiplane calcium imaging in a virtual track running task found a larger fraction of locomotion-activated somatostatin (SOM)-expressing and parvalbumin (PV)-expressing interneurons and a small fraction of the immobility-activated similar interneuron subtypes.^74^ Furthermore, in their follow-up study, they elucidated that the activity of both CA1 SOM- and PV-expressing interneurons during a goal-directed spatial navigation task was at first strongly suppressed upon exposure to a new environment but recovered as mice grew accustomed to it.^75^ The modulation of activity of SOM-expressing interneurons was context-independent, and their recovery from the suppression was blocked when behavioral recovery was prevented.

Pyramidal neuron disinhibition is provided by the interneuron-specific interneurons that express the vasoactive intestinal polypeptide (VIP) and selectively innervate other inhibitory interneurons. Calcium imaging of CA1 VIP-expressing interneurons suggests that their activity was modulated by velocity during locomotion and by reward and location during goal-oriented learning.^76^ Inhibition of VIP-expressing interneurons showed that disinhibition mediated by this interneuron subtype is necessary for reward learning and goal-directed enrichment of place cells, demonstrating that their behavioral state- and task demand-dependent disinhibition supports goal-oriented learning.

The relationship between various interneuron activities and electrical network activity in the hippocampus was shown via a two-photon calcium imaging study combined with LFP recordings. Two-photon calcium imaging of VIP-expressing interneurons and LFP recording in awake locomoting mice revealed their significant speed modulation and preferential recruitment during theta-run epochs but not during ripples.^77^ Moreover, fast 3D acousto-optical imaging of the basal dendrites of the PV-expressing interneurons combined with ipsilateral LFP field recording in awake mice visualized regenerative calcium spikes initiated at variable hot spots and propagated in both directions.^78^ During SWR doublets within a short temporal window, a supralinear dendritic summation emerged, suggesting their role as a dendritic spatiotemporal coincidence detector.

The dynamics of large-scale interneuron populations from the stratum oriens/alveus border to the stratum lacunosum moleculare in CA1 during navigation and learning was characterized via an acousto-optic deflector-based 3D two-photon functional imaging and post hoc immunohistochemistry.^37^ Identification and characterization of six molecularly defined interneuron subtypes indicated that a subset of CA1 interneurons exhibit spatially-tuned activity. Moreover, prominent reward modulation of SOM-expressing interneuron activity during goal-directed spatial learning was demonstrated. These and other findings collectively yield important insights into the functional organization of local inhibitory circuits.

#### 3.1.3 Astrocytes

Calcium dynamics in astrocytes influence nearby neuron and synapse activity, and also affect behavior. Two-photon calcium imaging revealed that hippocampal astrocytes responded to an anxiogenic environment through intracellular calcium activity, reflecting a mouse’s affective state during a virtual elevated plus maze task.^80^ Optogenetic activation that increases astrocytic intracellular calcium elicited anxiolytic behaviors, which was mediated by a mechanism where ATP released from activated astrocytes increased excitatory synaptic transmission in dentate granule cells.

During virtual navigation, concurrent two-photon calcium imaging of CA1 astrocytes and neurons demonstrated that astrocytes display calcium events carrying spatial information in topographically organized cellular subregions.^81^ Astrocyte-encoded spatial information was complementary and synergistic to that carried by neurons, which improved decoding when astrocytic signals were considered. A recent study further demonstrated that hippocampal astrocytes not only convey spatial information but also encode reward location.^82^ Chronic imaging of calcium activity of CA1 astrocytes during virtual navigation demonstrated that they display experience-dependent, persistent ramping activity toward the reward location in a familiar environment, with which the mouse trajectory was sufficiently decoded.

#### 3.1.4 Alzheimer’s disease and other disease models

Alzheimer’s disease (AD), a progressive neurological and neurodegenerative disorder, primarily affects the temporal lobe including the hippocampus, and is characterized by cognitive deficits and memory impairment. Hence, hippocampal cellular resolution imaging of AD mouse models can inevitably contribute to deepening our understanding of its pathophysiology. A study that imaged spontaneous CA1 pyramidal neuron activity in anesthetized amyloid precursor protein (APP)/presenilin-1 (PS1) transgenic AD model mice observed increased fractions of silent and hyperactive neurons near amyloid-β (Aβ) plaques stained with thioflavin-S in 6–7-month-old mice.^85^ Interestingly, the increase in hyperactive cells was already present before plaque formation in 1–2-month-old mice, and direct application of soluble Aβ elicited neuronal hyperactivation in wild-type mice, suggesting the crucial role of soluble Aβ in the early functional impairment in AD.

The oriens-lacunosum-moleculare (O-LM) interneurons is a type of SOM-expressing interneurons that controls CA1 pyramidal neuron activity. *In vivo* two-photon imaging of GFP-labeled O-LM neurons in APP/PS1 transgenic AD model mice revealed progressive axon loss and structural plasticity impairment of dendritic spines.^86^ Fear learning-induced, cholinergic input-dependent spine gain was also affected. Moreover, in these mice, calcium imaging demonstrated decreased O-LM interneuron responses to aversive stimulation. Thus, structural and functional imaging of this interneuron subtype identified impaired local circuit function and remodeling associated with memory impairment in AD.

Poll et al. used mice acquired from crossing of c-fos promoter GFP transgenic mice with APP/PS1 transgenic AD model mice and imaged the dynamics of their GFP-expressing CA1 pyramidal neurons over several days after contextual fear conditioning to assess the neuronal correlates of memory trace, referred to as an “engram,” in AD model mice.^87^ Learning-induced GFP expression and partial reactivation of engram cells during recall is preserved in AD mice. However, additional ensembles that encoded novel contextual information interfered with the intact engram and memory recall, indicating that memory trace interference is a key process that underlies memory deficits in AD.

To better comprehend memory deficits and associated hippocampal dysfunction in AD, it is vital to explore hippocampal representations of spatiotemporal information during behavior and learning. Chronic calcium imaging of the CA1 pyramidal neurons in App knock-in AD model mice navigating a virtual environment showed that place cells were preferentially and progressively reduced near Aβ aggregates, whereas time cells remained unaltered.^47^ These results reveal the differential impact of Aβ aggregates on two major modalities of episodic memory.

To date, only a few studies have examined abnormal hippocampal neural circuit activity in mouse models of brain disorders other than AD using two-photon imaging. Calcium imaging of an awake, chronic mouse model of temporal lobe epilepsy demonstrated that GABAergic neurons are preferentially recruited during spontaneous interictal activity in the CA1 region.^79^ A study investigated a neural substrate of cognitive deficits in $\mathrm{Df}(16{)}^{+/-}$ mice, a mouse model of 22q11.2 deletion that is a common genetic risk factor for cognitive dysfunction and schizophrenia, via two-photon calcium imaging of CA1 pyramidal neurons.^88^ This revealed a significant deficit in goal-oriented learning and reduced stability and a lack of reorganization of place cell maps toward the goal location. Another study imaged the formation and plasticity of a CA1 place cell map during spatial learning using VR in a mouse model of autism spectrum disorder (ASD) that lacks the synaptic scaffold protein Shank2.^45^ The Shank2-deficient ASD model mice ran more, received more rewards, and exhibited more enhanced goal-directed behavior than the wild-type mice. Interestingly, in the CA1 of these mice, the learning-induced over-representation of landmarks was absent, while the over-representation of rewards was substantially increased. This indicates that abnormally distorted hippocampal cognitive mapping may underlie cognitive and behavioral abnormalities in a subset of ASDs. Altogether, these findings collectively indicate that while a mouse model of 22q11.2 deletion syndrome shows deficits in hippocampal reward over-representation and goal-oriented learning,^88^ a specific deletion of the *Shank2* gene leads to substantially augmented reward over-representation and enhanced goal-directed behavior.^45^ The hippocampal reward representation and related behavior can thus be altered bidirectionally in mutually opposite directions in different mouse models of neurodevelopmental disorders.

### 3.2 Dentate Gyrus

#### 3.2.1 Granule cells and mossy cells

The dentate gyrus (DG) is located deeper than CA1 and is thus technically more difficult to image. One of the early studies imaged dentate granule cell activity in awake mice via an imaging cannula that left the hippocampus intact.^98^ Granule cells were labeled with the red indicator R-CaMP1.07 and imaged 600 to 800  μm below the hippocampal CA1 surface using 1040-nm excitation, although they could also be imaged using the green indicator GCaMP6s with 920-nm excitation. Individual granule cell activity patterns were generally sparse, were heterogeneously associated with locomotion, and varied flexibly across days.

Another early study replaced a portion of the cortex and CA1 with an imaging cannula to image the activity of GCaMP6f-labeled adult-born granule cells (abGCs) and mature granule cells (mGCs).^99^ abGCs exhibited higher activity and less spatial tuning than mGCs. Optogenetic silencing of abGCs showed their active participation in contextual encoding and discrimination. Moreover, abGCs and mGCs were differentially recruited to interictal epileptiform discharges in a mouse model of chronic epilepsy.^100^ The same group used this technique further to contrast the activity of mossy cells, another major excitatory principal cell type, in the hilus of the DG with granule cells.^101^ Their characterization showed that mossy cells are more active than granule cells, exhibited significant but more diffuse spatial tuning than granule cells, and discriminated contexts through robust remapping.

In the DG, granule cells transform information from the entorhinal cortex into a sparse output. Chronic imaging of neuronal activity in the DG, CA3, and CA1 revealed that granule cells in the DG encode stable spatial information over multiple days,^102^ whereas pyramidal neurons in the CA1 and CA3 exhibit highly context-specific and constantly changing spatial representations. A follow-up study by Cholvin and Bartos examined whether left-right lateralization in spatial and contextual coding properties existed in granule cells.^103^ They did this by imaging the activity of granule cells in the left or right DG. Despite similar mean activity levels in both hemispheres, the granule cells in the left DG displayed higher spatial tuning, higher across-day stability, less run-to-run variability, and greater remapping, implying that contextual discrimination and contextual generalization is higher in the left and right DG, respectively.

The activity of GCaMP6s-labeled granule cells and jRGECO1a-labeled inputs from the medial entorhinal cortex was imaged via the dual-color *in vivo* two-photon calcium imaging.^104^ This study revealed sparse and synchronized activity of the dentate granule cell populations associated with entorhinal cortex activation in immobile mice. These events incorporated place- and speed-coding granule cells, and these cells were responsive to environmental changes. Furthermore, they were significantly similar to population activity patterns during locomotion.

Imaging of the dentate granule cell activity in mice running on a cue-manipulated treadmill identified robust sensory cue responses in these cells.^105^ Such “cue cell” responses are stimulus-specific, more stable than place cell responses, and are associated with the suppression of neighboring cells. This suggests that the DG performs parallel processing of spatial and sensory cue information during the first stage of hippocampal information processing.

#### 3.2.2 Neurogenesis and astrogenesis

The mechanisms of neurogenesis and astrogenesis in the DG were also explored using two-photon microscopy. Chronic *in vivo* two-photon imaging was used to follow genetically labeled neural stem cells (NSCs) and their progeny in the mouse hippocampus.^106^^,^^107^ The studies revealed that achaete-scute homolog 1 (Ascl1)-targeted NSCs underwent limited proliferative activity before they terminally differentiate, whereas a fraction of GLI family zinc finger 1 (Gli1)-targeted NSCs displayed long-term self-renewal in the adult hippocampus.

Dentate astrocytes were deemed to derive from perinatal and adult radial glia-like NSCs (rNSCs). However, a study that assessed the generation and proliferation of astrocytes in the DG across the lifespan identified that the predominant source of newborn astrocytes in the adult DG are not rNSCs, but are locally dividing astrocytes in neurogenic niches.^108^ In this study, intravital two-photon imaging of RFP-labeled astrocytes in the adult hippocampus encompassing the dorsal CA1 to the DG showed that astrocytes nearby the hippocampal fissure generated a few astrocytes through local proliferation.

### 3.3 Dendrite Imaging

A decade after the first structural imaging of GFP-labeled dendritic spines in the CA1 in living mice,^6^ chronic two-photon imaging of YFP-labeled CA1 pyramidal neurons over weeks demonstrated that the dendritic spines of CA1 pyramidal neurons displayed structural plasticity in adult mice.^90^ This study demonstrated that although the spine density in the stratum radiatum was overall stable, a small percentage of spines appeared and disappeared when observed at 4-day intervals. More recently, two-photon super-resolution stimulated emission depletion (2P-STED) microscopy showed a twofold increase in spine density than reported by conventional 2P microscopy and a high spine turnover rate of about 40% within 4 days.^36^ This indicates that the high density and short necks of the hippocampal pyramidal cell spines make them difficult to be imaged precisely, a limitation that can be overcome by sophisticated high-resolution microscopy to image them as accurately as possible.

The first imaging of pyramidal cell dendrite activity during virtual navigation was conducted using ETL-mediated multiplane two-photon calcium imaging of CA1 pyramidal neurons that co-acquired images of their soma and dendrites.^9^ This study uncovered spatiotemporally variable regenerative dendritic branch spiking events in hippocampal place cells and revealed that the prevalence of these events predicts the spatial precision and stability of place fields. Their follow-up study further imaged somatic and dendritic activity during virtual navigation in familiar and novel environments^91^ and found that the occurrence of local dendritic spikes preceded firing of the somatic place field. It was also found that the emergence of somatic place-field firing was associated with reduced dendritic inhibition and increased somatic inhibition, as shown by calcium imaging of axons of SOM- and PV-expressing interneurons, respectively. To further image excitatory synaptic inputs associated with place-field computation, this group conducted imaging using iGluSnFR, a membrane-targeted genetically encoded glutamate sensor, and found that spatially tuned excitatory inputs to place cells are temporally coactive and anatomically clustered along the dendrites.^48^ Altogether, these series of studies have provided remarkable insights into the dendritic and synaptic mechanisms underlying place-field formation.

Rolotti et al.^92^ labeled CA1 pyramidal cells with GCaMP via *in utero* electroporation and imaged their somatic and basal dendritic activity through high-speed scanning along a Z-axis. Concurrent imaging with LFP recording revealed that the basal dendrites of CA1 pyramidal cells were coactive with their cell bodies during SWR and found that this somatic-dendritic coupling is differently modulated by behavior and network states.

Apart from these studies, calcium imaging of basal dendrites of PV-expressing interneurons^78^ and structural imaging of dendritic spine morphology of O-LM neurons in AD model mice^86^ have been reported.

### 3.4 Axon Imaging

The medial septum provides GABAergic input to hippocampal interneurons. A study conducted functional imaging of septo-hippocampal GABAergic axonal boutons in hippocampal CA1 of behaving mice.^8^ Bouton populations were activated during locomotion and salient sensory stimuli, with their responses increasing with stimulus intensity. The observed patterns of bouton activity were primarily determined through presynaptic spiking of septal GABAergic neurons.

Lee et al. conducted imaging on axon terminal activity of PV-expressing basket cells in the superficial and deep pyramidal cell sublayers in the dorsal CA1.^93^ Running evoked preferential activation of their axon terminals around the somata of pyramidal cells in the deep sublayer. Together with the findings obtained from *in vitro* paired recordings and computational modeling, the results revealed that PV-expressing basket cells exert greater inhibition in CA1 pyramidal neurons located in the deep compared to the superficial sublayer of the stratum pyramidale, revealing the presence of heterogeneous inhibitory microcircuits in CA1.

The hippocampus receives a variety of neuromodulatory inputs from each originating nucleus. Serotonergic projection activity from the median raphe nucleus to the CA1 hippocampus was imaged in mice behaving in VR.^94^ They identified two distinct subsets of the projections: one linked to reward delivery and the other to locomotion. Local optogenetic manipulation of these fibers modulated reward-induced behavior. Another study imaged axon activity from the locus coeruleus (LC) to the dorsal CA1 for several days during spatial learning.^95^ The LC axons exhibited increased activity near a new reward location upon translocation of the reward. Photoactivation of the LC axons induced place cell over-representation near the reward, while photoinhibition suppressed it, suggesting that the LC axons signal reward translocation to CA1 to induce place cell reorganization during goal-directed spatial learning.

Cholvin et al.^96^ imaged axonal inputs of the medial entorhinal cortex to the CA1, CA3, and DG in mice exploring two dissimilar virtual environments and found that the MEC inputs to each subfield show different spatial coding properties. MEC inputs remap even more strongly between environments than hippocampal principal cells, while the spatially tuned responses of hippocampal principal cells were more reliable and stable than those of the MEC inputs, demonstrating that the dynamic spatio-contextual information coded by the MEC inputs are transformed into stable spatial maps in the hippocampus. On the other hand, two-photon calcium imaging of CA3 axonal projections to CA1, referred to as Schaffer collaterals, in mice navigating on a belt treadmill with or without spatial sensory cues demonstrated that CA3 projections that convey behaviorally relevant sensory information were selectively recruited during the memory replay events, whereas axons encoding uninformative sensory cues were adaptively inhibited during SWRs,^97^ indicating that recruitment or exclusion of cue-driven CA3 axonal activity determined by the behavioral relevance of the sensory cues likely acts as a mechanism that adaptively filters sensory experience during memory consolidation.

## 4 Conclusions and Future Perspectives

**Fig. 5:**
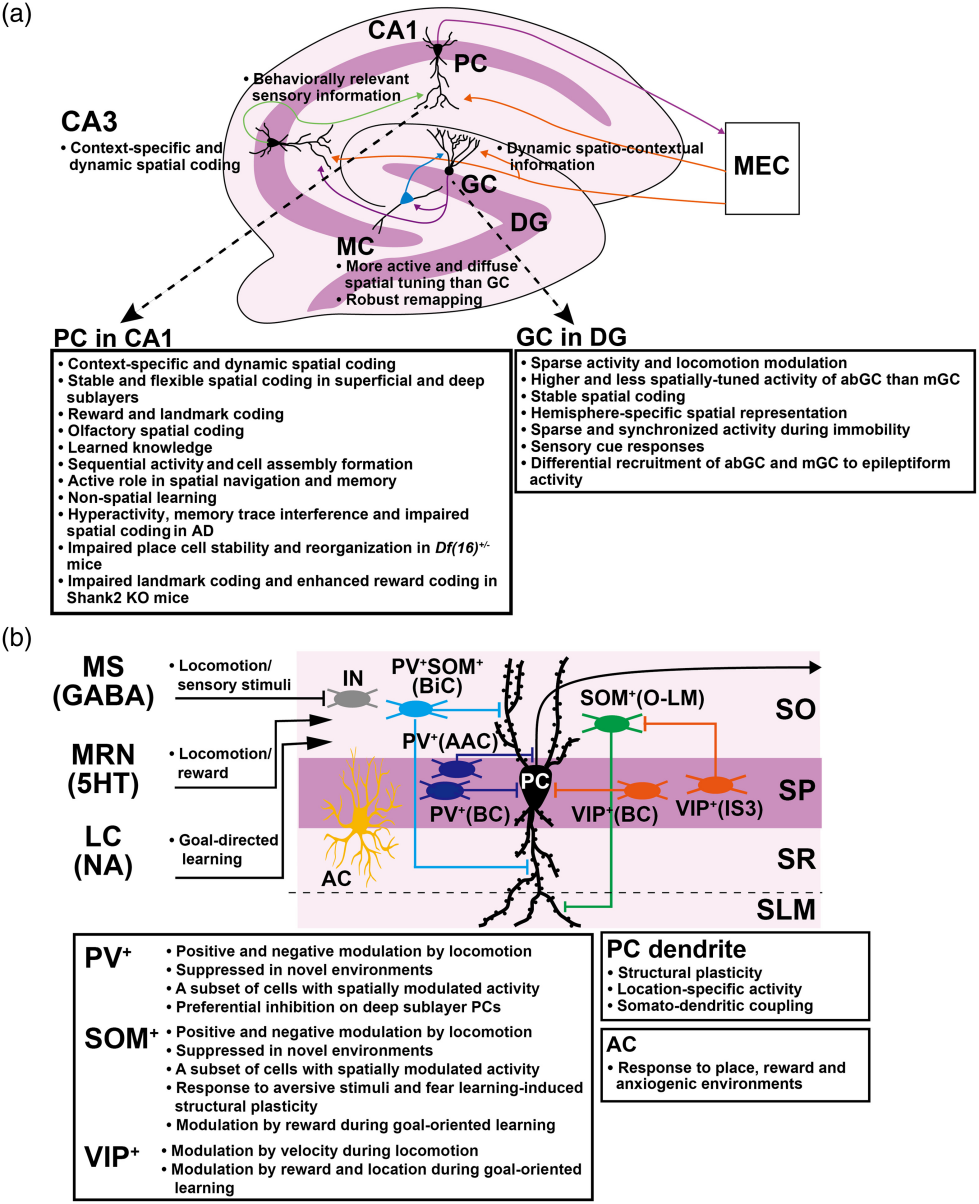
Summary of findings on the function of hippocampal neural circuits obtained by multiphoton imaging. (a) Entorhinal cortex-hippocampal tri-synaptic circuit. (b) CA1 local circuit. See text for details. AAC, axo-axonic cells; abGC, adult-born GC; AC, astrocytes; AD, Alzheimer’s disease; BC, basket cells; BiC, bistratified cells; DG, dentate gyrus; GABA, gamma-aminobutyric acid; GC, granule cells; IN, GABAergic interneurons; IS3, type 3 interneuron-specific cells; KO, knockout; LC, locus coeruleus; MEC, medial entorhinal cortex; mGC, mature GC; MRN, median raphe nucleus; MC, mossy cells; MS, medial septum; NA, noradrenaline; O-LM, oriens-lacunosum-moleculare cells; PC, pyramidal cells; ${\mathrm{PV}}^{+}$, parvalbumin-positive; SLM, stratum lacunosum-moleculare; SO, stratum oriens; ${\mathrm{SOM}}^{+}$, somatostatin-positive; SP, stratum pyramidale; SR, stratum radiatum; SWR, sharp-wave ripple; ${\mathrm{VIP}}^{+}$, vasoactive intestinal polypeptide-positive; and 5HT, serotonin.

This review highlighted the technology of and biological findings obtained from multiphoton imaging of hippocampal neural circuits. More than a decade of intense research has been conducted since the first place cell imaging.^7^ Since then, multiphoton imaging has revealed extensive detail regarding the region-, cell-type-, and pathway-specific functions of hippocampal neural circuits (Fig. 5). At the level of the entorhinal cortex-hippocampal circuit [Fig. 5(a)], dynamic spatio-contextual inputs from the MEC are transformed into stable maps in the hippocampus. While CA1 forms more context-dependent and dynamic maps, granule cells in the DG exhibit relatively sparse activity and more stable map than CA1. CA1 serves as the most easily observed hippocampal model circuit, and its involvement in spatial and non-spatial coding, various forms of learning, and brain disorders has been studied in great detail. On the other hand, DG has the unique feature that there are two populations of granule cells (abGC and mGC), and another excitable cell type (mossy cells). The function of these cell types is gradually being elucidated by taking advantage of multiphoton imaging’s ability to genetically label and directly observe specific cell populations.

In the CA1 circuit [Fig. 5(b)], different neuromodulator inputs are activated in response to locomotion, sensory stimuli, reward, and learning. Distinct functions of inhibitory interneurons expressing different molecular markers have also been identified. In particular, each subtype was found to be differentially modulated by locomotion, velocity, place, and reward in different tasks. The strength of imaging also lies in its ability to observe astrocytic responses to various factors and pyramidal cell dendritic activity. Findings in the CA1 local circuit were obtained primarily from the stratum oriens and stratum pyramidale, which are readily accessible by imaging. As deep imaging becomes more common, new insights into the CA1 local circuit function may be obtained through more imaging studies focusing on the stratum radiatum and stratum lacunosum-moleculare. Hippocampal interneurons are diverse, and cells expressing a single molecular marker are known to contain multiple subtypes. Future studies will require imaging combined with more specific classification methods such as multiplexed genetic labeling and/or post hoc immunostaining of multiple molecular markers.

To summarize the above, we would like to elucidate some future prospects. First, there have been many cutting-edge imaging techniques developed to date, including those that were not covered in detail in this review, such as fast and high-resolution volumetric imaging^109^^,^^110^ and large-scale two-photon imaging in freely moving animals.^111^ As these microscopes and imaging techniques continue to be developed, more and more new findings will be obtained through finer and more large-scale observations.^112^ Second, in addition to genetically encoded calcium indicators, many other fluorescent sensors for different neurotransmitters have been developed.^21^ However, most of them have not yet been applied to hippocampal research. Therefore, the application of these biosensors will provide insight into their dynamics in the hippocampus during cognition and behavior. Finally, further research using mouse models of AD and other understudied brain disorder models will contribute to a deeper understanding of the function and dysfunction of this unique and intricate brain region in health and disease.^113^

## Data Availability

Code, data, and material sharing are not applicable to this review, as none of them were created or analyzed.
